# Inertial biometry from commercial 3D body meshes

**DOI:** 10.1242/bio.058927

**Published:** 2022-03-28

**Authors:** Alberto E. Minetti, Luca Ruggiero

**Affiliations:** Department of Human Physiology, Faculty of Medicine, University of Milan, Via Mangiagalli 32, 20133 Milan, Italy

**Keywords:** Inertia, 3D mesh, Man, Woman, Horse, Cat

## Abstract

Body segments inertial parameters (or, more generally encompassing humans and animal species, inertial biometry), often necessary in kinetics calculations, have been obtained in the past from cadavers, medical 3D imaging, 3D scanning, or geometric approximations. This restricted the inertial archives to a few species. The methodology presented here uses commercial 3D meshes of human and animal bodies, which can be further re-shaped and ‘posed’, according to an underlying skeletal structure, before processing. The sequence of steps from virtually chopping the mesh to the estimation of inertial parameters of body segments is described. The accuracy of the method is tested by comparing the estimated results to real data published for humans (male and female), horses, and domestic cats. The proposed procedure opens the possibility of remarkably expanding biomechanics research when body size and shape change, or when external tools, such as prosthesis and sport material, take part in biological movement.

## Abstract (precis)

**Summary:** Using commercial digital 3D body meshes, body segment inertial parameters of humans and animals can be estimated, expanding the possibilities and the spectrum of species considered in biomechanics research.

## INTRODUCTION

The diffusion of optoelectronic analysis systems has expanded the kinematic and kinetic research spectrum. In parallel, there is a growing need for body segment inertial parameters (BSPs; or, more generally encompassing humans and animal species, inertial biometry) to move from the ‘standard’ man to a variety of other body shapes, as its accuracy is well known to affect modelling (e.g. Yeadon, 1990) and kinetic outcomes (Nguyen and Reynolds, 2014; Fritz et al., 2019). Starting from men (Dempster, 1955) and women (Young et al., 1983), BSPs of infants and children (Schneider and Zernicke, 1992; Ganley and Powers, 2004), overweight and obese subjects (Chambers et al., 2010; Merrill et al., 2019) and the elderly (Pavol et al., 2002) have been increasingly researched in the last two decades. Animal biomechanics is no different: studies have determined inertial biometry for ergonomic reasons in specific animals (e.g. German Shepherd for the Royal Canadian Mounted Police; Jones et al., 2018), in breeds of the same subspecies (hot-, warm-, and cold-blooded horses; Buchner et al., 1997; Nauwelaerts et al., 2011), or for evolutionary purposes from dinosaurs (Bates et al., 2009) to our closest ancestors (Pan troglodytes; Schoonaert et al., 2007).

The search for standard BSPs started at the end of the 19th century, with Braune and Fisher (1890, 1892), followed by Weinbach (1938), Dempster (1955, 1959; reported in table form in the book by Winter, 1979), Santschi et al. (1963), Clauser et al. (1969), and Chandler et al. (1975), mostly with the study of human cadavers. However, as cadavers are not always available, other techniques have been proposed to determine BSPs for specific populations, such as gamma camera (Zatsiorsky et al., 1990), magnetic resonance imaging (MRI; Mungiole and Martin, 1990) and dual energy X-ray absorptiometry (DXA; Durkin et al., 2002). The use of these methods can be very expensive and not entirely practical for pregnant women, severely obese patients, and big animals.

In an attempt to solve these issues, geometric methods have been developed, whereas BSPs can be determined using the respective volume (e.g. Jensen, 1978; Yeadon, 1990; Jensen et al., 1996) and average density values (Dempster, 1955; Buchner et al., 1997). Advancements in technology have simplified volumetric estimation with techniques such as digital photogrammetry (Nauwelaerts and Clayton, 2018), 3D depth camera (Kordi et al., 2019; Choppin et al., 2021) or laser scanning (Rossi et al., 2013). However, these procedures may still be expensive, or may be impractical when specific animal species, body postures, or bodies with added tools are studied.

For their own needs, motion pictures industries produce 3D meshes of humans and animals, using either 3D scanning or digital reconstructions resembling the desired body. Examples can be found at the websites: Free3D (https://free3d.com), TurboSquid (www.turbosquid.com/3d) and Poser (https://www.posersoftware.com), to name a few. The last product, besides providing high-resolution 3D meshes of humans and some animals, allows the user to move limbs according to an internal ‘virtual’ skeleton creating new ‘poses’, and interaction between meshes. The resulting mesh can then be cut into 3D segments through programs such as Cheetah3D (www.cheetah3d.com), and BSPs calculated for example with AutoCAD (www.autodesk.co.uk) or Rhino3D (www.rhino3d.com).

From all the above considerations, the aim of this research was to use commercially available 3D meshes and edit them with the available programs to determine inertial biometry of humans, horses, and domestic cats. As those meshes have been manufactured to accurately represent a 3D body shape, we could expect to estimate ‘realistic’ BSPs from them. The proposed method starts from a 3D mesh of the entire body (human male and female, horse, and domestic cat), chops it into segments, calculates BSPs, and compares them to the gold-standard values of Dempster (1955), Buchner et al. (1997), and Hoy and Zernicke (1985) for humans, horses, and the domestic cat, respectively. The software used represents just a ‘working’ suggestion.

## RESULTS

Table 1 contains BSPs estimated with the present method for the ‘standard’ male and female bodies, compared to Dempster's reference data (in Winter, 1979), and for females only compared to reference data from Merrill et al. (2019). Ratios between each estimated and reference value have been reported in the ‘Ratio’ column, along with the mean, standard deviation (s.d.), and coefficient of variation (CV) across all segments.

**Table 1.**
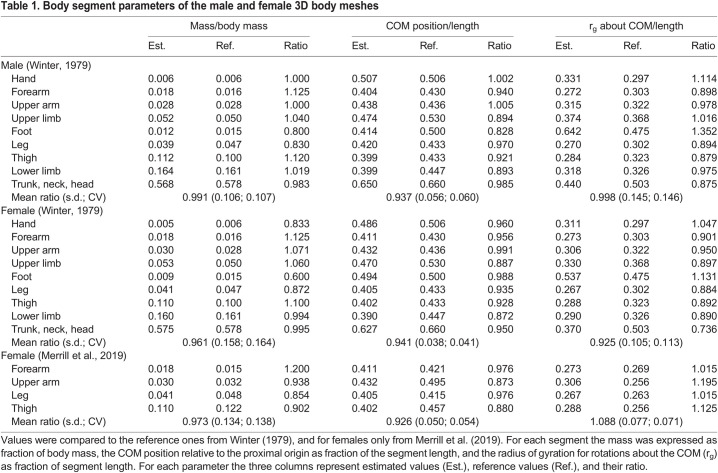
Body segment parameters of the male and female 3D body meshes

BSPs estimated from the horse and domestic cat meshes are shown in Table 2. Here the results are compared with reference horse and domestic cat data by Buchner and colleagues (1997), and by Hoy and Zernicke (1985), respectively. Notably, the fractional mass of all body segments and the centre of mass (COM) position (as fraction of segment length) from only four body segments were available for the domestic cat in Hoy and Zernicke (1985).

**Table 2.**
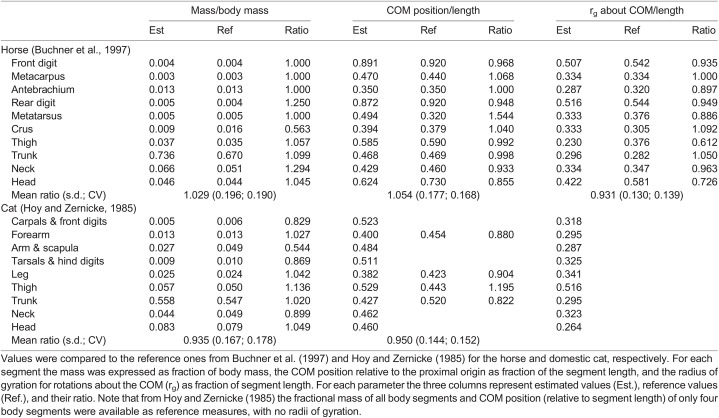
Body segment parameters of the horse and domestic cat 3D body meshes

**Fig. 1.**
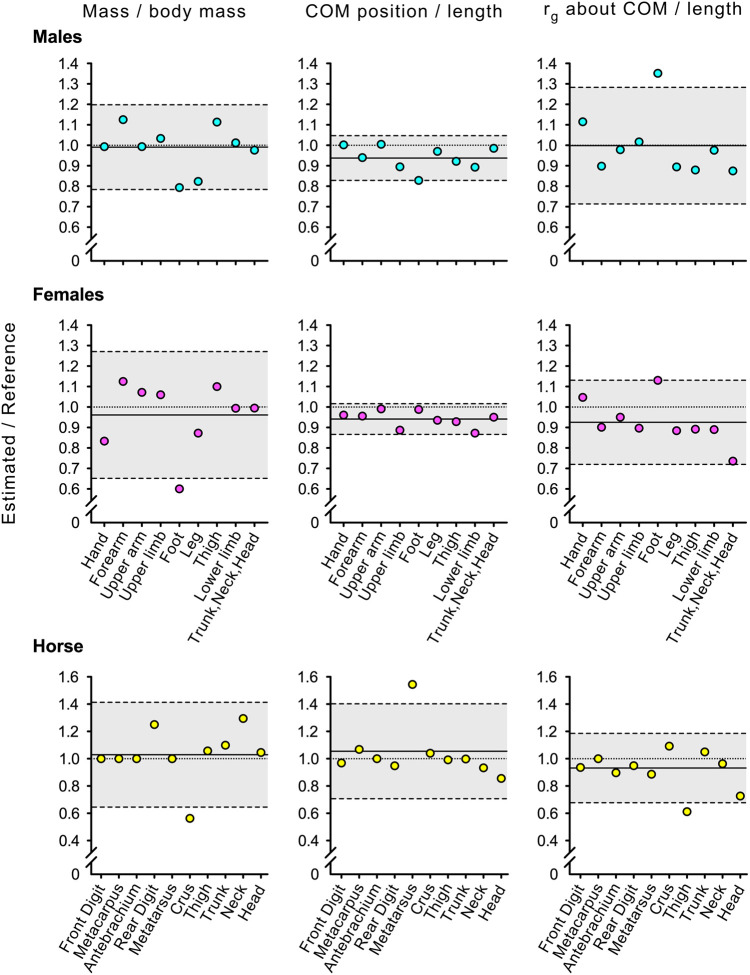
**Ninety-five percent limits of agreement (LoA) of ratios between estimated and reference measures.** The solid line represents the mean difference, whereas the dashed lines enclosing the grey area represent upper and lower limits with 95% confidence. The dotted reference lines at 1 are included to frame the data relative to the scenario of no mean difference between measures. Top row: males (cyan); middle row: females (magenta); bottom row: horse (yellow). Left column: mass expressed as fraction of body mass; middle column: centre of mass (COM) position relative to the proximal origin as fraction of the segment length; right column: radius of gyration for rotations about the COM (r_g_) as fraction of segment length. Reference measures were from Winter (1979) for males and females, and from Buchner et al. (1997) for the horse.

**Fig. 2.**
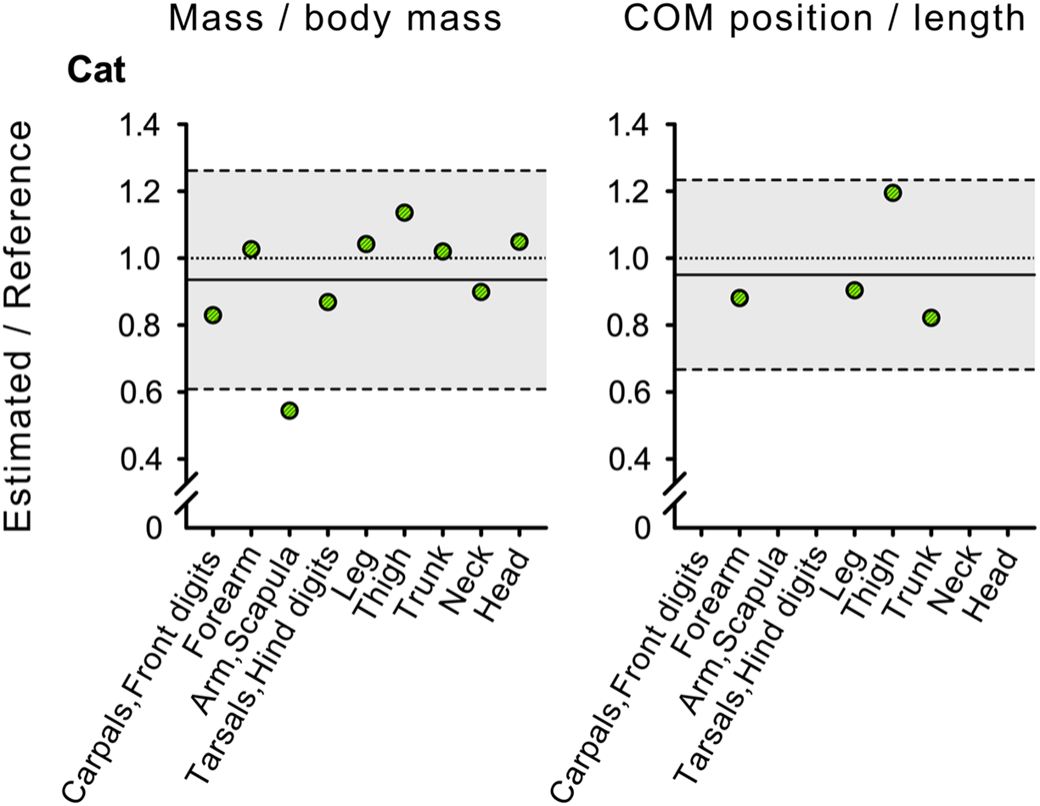
**Ninety-five percent LoA of ratios between estimated and reference measures for the domestic cat (*Felis catus*).** The solid line represents the mean difference, whereas the dashed lines enclosing the grey area represent upper and lower limits with 95% confidence. The dotted reference lines at 1 are included to frame the data relative to the scenario of no mean difference between measures. Left column: mass expressed as fraction of body mass; middle column: COM position relative to the proximal origin as fraction of the segment length. Reference measures were from Hoy and Zernicke (1985). The fractional mass of all body segments and COM position (relative to segment length) of four body segments were available as reference measures. Thus, only four values for COM position are reported in the figure, with no radii of gyration.

Comparisons of estimated and reference BSPs are also graphically reported through 95% LoA of their ratios in Fig. 1 for males, females, and the horse, and in Fig. 2 for the domestic cat.

For the inter-rater reliability analysis, ICC_2,1_ was 0.99, 0.94, and 0.97 for fractional mass, COM position, and radii of gyration, respectively (95% confidence intervals: 0.98–1.00, 0.89–0.99, and 0.95–0.99, respectively). All other parameters characterising both inter- and intra-rater reliability are reported in Table 3.

**Table 3.**
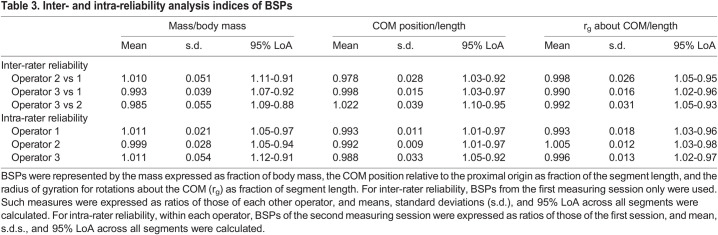
Inter- and intra-reliability analysis indices of BSPs

## DISCUSSION

The match between estimated and reference BSPs from the male and female 3D mesh is quite consistent, as witnessed by the ‘Ratio’ columns in Table 1, and by the LoA in Fig. 1. The greatest deviations are regarding the foot, a segment whose accuracy in mesh design can be expected to be lower than others. Additionally, while the foot in Winter (1979) is considered from the ankle centre to the 2nd metatarsal head, in the present research all segment volume was included, as such points would be hard to identify on the mesh. This would also explain the greatly overestimated radius of gyration about the COM relative to reference values. Notwithstanding, the ‘by-eye’ procedure of cutting the 3D mesh generated data remarkably close to reference values (Winter, 1979; Merrill et al., 2019).

The same comments apply to the BSPs from the horse 3D mesh (Table 2 and Fig. 1). The match between estimated and reference values is weaker than in humans. Differently from Buchner et al. (1997), in the chopping process the shoulder was considered part of the trunk, which may explain the overestimation in segment mass and radius of gyration compared to reference values. For horses, the diversity of breeds can considerably affect BSPs (Nauwelaerts et al., 2011). While in Buchner et al. (1997) warm-blooded horses (intermediate morphotype between ‘hot-’ and ‘cold-blooded’ ones) were considered, the horse mesh morphotype in the present manuscript may have been more gracile, which may explain the considerably lower fractional mass of the crus and estimated radii of gyration for the antebrachium and thigh compared to the reference ones, and the weaker overall agreement between methods compared to humans.

Although fractional masses for all body segments, COM positions for only four body segments, and no radii of gyration (as fraction of segments length) were available from Hoy and Zernicke (1985), observed values relative to reference ones were also close also in the domestic cat. Notably, the BSPs with the greatest deviations from reference values were the fractional mass of arm and scapula, and the COM position of the thigh. These are the hardest segments to identify in the chopping process, as the corresponding limb merges with the trunk. The domestic cat (*F. catus*) has been specifically included in the present manuscript because it is of particular interest as it belongs to the Felidae, a family of mammals that presents postural and geometric similarity across masses up to 200 kg (Day and Jayne, 2007; Dick and Clemente, 2017).

The methodology illustrated in this paper comes with pros and cons. The main advantage is that cadavers (e.g. Buchner et al., 1997), radiative imaging (Zatsiorsky et al., 1990; Mungiole and Martin, 1990; Durkin et al., 2002), or whole-body 3D scans are not necessary. This may be especially useful in animal studies, where obtaining inertial biometry is troublesome, usually done through cadavers (e.g. Crompton et al., 1996; Buchner et al., 1997), geometric methods from anaesthetised animals (e.g. Schoonaert et al., 2007), 3D slicing from sagittal and frontal profiles (e.g. Henderson, 1999), 3D scanning and computer modelling from available or reconstructed skeletons (e.g. Bates et al., 2009). When studying animal biomechanics, if BSPs are inaccessible, and none of the above-mentioned procedures are readily feasible, the methodology of this paper could be used without the risk of reaching incorrect conclusions.

Another advantage is that the methodology of the present paper is applicable to the whole spectrum of needs (e.g. specific body postures, big animals, less commonly studied species). The present research extends Alexander's technique (Alexander, 1989). In that case, the body COM was located by suspending a plastic model of the animal after drilling a hole in proximity to where the lungs should be. Inertial parameters for trunk and segments of species could then be determined. Accordingly, with the methodology herein proposed, a 3D laser scan of the plastic model of a species, or a digital mesh, and some post-processing would be enough to allow estimation of BSPs. Moreover, from a body mesh, the position of the whole body, the overall shape of limbs or trunk could be altered to follow a given sagittal or frontal profile, or specific body measures. For example, in sports biomechanics the proposed procedures could be used in diving, gymnastics, or figure skating to estimate the radii of gyration of the whole body in specific aerial positions. Alternatively, in medicine and healthcare, dimensions of BSPs could be altered to represent different stages of pregnancy or bed rest-induced atrophy.

There are drawbacks, though, to this technique: (1) unless regional densities are available, the same density is attributed to the entire body; (2) air cavities volume is approximated considering vital capacity, and lungs and airways are approximately shaped through ellipsoids and positioned; (3) mesh cutting is guided by visual clues, not by recognition of internal anatomical structures; (4) misalignment between the segment coordinate system (kinematically meaningful) and chosen coordinate reference system of chopped segments. Many of these problems can be attenuated by careful and sensible planning of the described pipeline procedures, as witnessed by the reported agreement between estimated and reference BSPs.

As highlighted earlier (see INTRODUCTION), the software used herein represent just a working suggestion, and other software could be used to accomplish the same results. For example, Boolean operations on meshes could be performed with freeware such as Blender (https://www.blender.org) or MeshLab (https://www.meshlab.net), whereas programs other than Rhino 3D could be used for volumetric measures and to estimate BSPs.

In conclusion, the presented methodology is meant to expand the archive of inertial parameters for segments and bodies not immediately available for dissection. Also, it allows data to be obtained for variations of body shape as generated, for example, by pregnancy, prosthetics, specific body postures, or for different animal species without the need of invasive measures, in a low-cost and consumer-friendly manner. In addition, with the increasing accessibility and portability of scanning techniques such as Lidar (e.g. https://www.apple.com/iphone-12-pro/), objects' mesh can potentially be easily acquired, and subsequently chopped or modified similar to the procedures presented in this research.

## MATERIALS AND METHODS

**Fig. 3.**
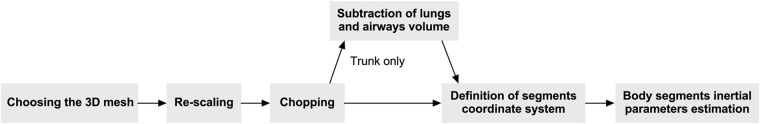
Workflow of the procedures, from choosing the 3D mesh to the estimation of BSPs.

The procedures, from choosing the 3D mesh to estimation of BSPs, are reported in detail below. Fig. 3 depicts the corresponding workflow.

### Choosing the 3D mesh

We used Poser (version 11.0) and four of the provided meshes: Homme (male), Femme (female), Horse, and Cat. The files were also exported to a CAD program (Rhino3D) to check their integrity (no holes in the mesh).

### Re-scaling

For the male and female body, by using the preliminary measurements of their volume, and with a general density of 1.05 g/cm^3^ (density weighted average from Winter, 1979; airways space not considered), we re-scaled them (Cheetah3D, version 7.5.1) to correspond to 1.77 and 1.69 m of height (74 and 58 kg of mass, respectively). These target heights were chosen in accordance with the average height of males and females in Martin et al. (1987), allowing to estimate lungs and airways volume to subtract to the 3D meshes (see section ‘Subtraction of lungs and airways volume from 3D meshes’ below). For the horse and domestic cat, with a general density of 1.05 g/cm^3^ (airways space not considered), the volume was scaled to achieve a body mass of about 500 g and 3 Kg, respectively. These values were within the body mass range in Buchner et al. (1997) and Minetti et al. (1999) for the horse, and in Watanabe and Frank (1975) and Hoy and Zernicke (1985) for the domestic cat, allowing us to estimate the respective lung and airway volume from Gehr and Erni (1980) and Watanabe and Frank (1975) (see section ‘Subtraction of lungs and airways volume from 3D meshes’ below).

### Chopping

**Fig. 4.**
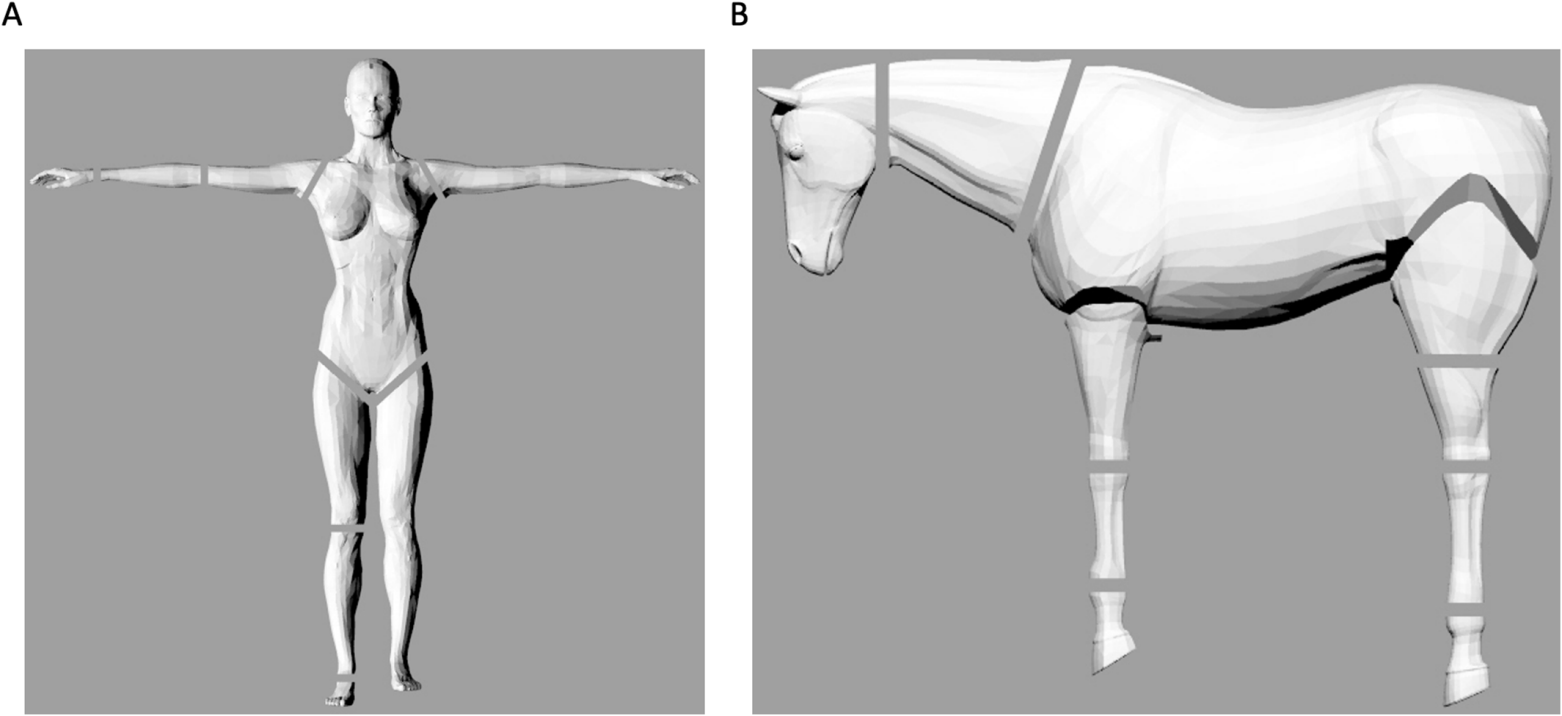
Examples of partitioning into segments of the 3D body meshes of a female (A) and horse (B).

To partition the entire 3D mesh into pieces it is necessary to use programs enabling Boolean operations between meshes (e.g. Cheetah3D). Single (very thin) planes (boxes) can be drawn to interact with the total body and extract single 3D segments (Fig. 4; a virtual chopped example of the horse is provided at https://skfb.ly/6XVuD). The boundaries between body segments were defined according to Dempster (1955; male and female), Buchner et al. (1997; horse), and Hoy and Zernicke (1985; cat). Obviously, the accurate set of the ‘cutting’ plane is crucial in obtaining reliable inertial data, which can be set differently for every application/movement.

### Coordinate system

To determine the position of the COM and moments of inertia, we defined the coordinate reference system for every segment (again, by using Cheetah3D). The origin was set to roughly correspond to the proximal joint centre, i.e. the middle of the proximal surface generated by the chopping process. The main axis was chosen as running along the major length of the segment, while the second one was chosen as to generate with the first axis the relevant (most significant) rotation plane in locomotion, i.e. the sagittal plane.

### Estimation

The single segments were then transferred to Rhino3D for the inertia parameters estimation (.stl file format). Segment mass (based on volumetric estimation and using a density of 1.05 g/cm^3^), COM position and the radius of gyration for rotations about the COM (r_g_) were then computed. For comparability with previous literature, and to account for the model volumetric scaling, for each segment the mass was expressed as fraction of body mass, while the COM position relative to the proximal origin and r_g_ for rotation about the COM as fraction of the segment length.

### Subtraction of lungs and airways volume from 3D meshes

The process described above was repeated for each segment, apart from the trunk, which needed some additional modifications to account for the lungs and airways space. Pairs of ellipsoids were scaled to collectively correspond to the average volume of male and female (5.4 and 4.3 l; Martin et al., 1987), horse (38 l; Gehr and Erni, 1980), and domestic cat (0.24 l; Watanabe and Frank, 1975) vital capacity, and placed symmetrically to be contained in the thorax. Then, a Boolean subtraction produced trunks with an air cavity within them for further processing. A similar procedure has previously been applied to account for lung space in other species (e.g. crocodiles; Henderson, 2003).

### Reliability analysis

To characterise inter- and intra-rater reliability for the procedures of the present manuscript, three different operators obtained BSPs (fractional mass, COM position, r_g_) from the 3D mesh of the male body (Homme, from Poser 11), repeating all the procedures mentioned above, twice. Body segments were identified according to Fig. 4A. Lungs and airways were placed in the thorax of the 3D mesh according to the details above (see section ‘Subtraction of lungs and airways volume from 3D meshes’). For inter-rater reliability, BSPs from the first measuring session only were used. Intraclass correlation coefficient (two-way random model, absolute agreement; ICC_2,1_) was calculated across all operators (SPSS software; version 27) for each parameter. Additionally, BSPs were expressed as ratios of those of each other operator, and means, standard deviations (s.d.), and 95% LoA (Bland and Altman, 1999) were calculated. For intra-rater reliability, within each operator, BSPs of the second measuring session were expressed as ratios of those of the first session, and mean, s.d.s., and 95% LoA were calculated.
